# Direct measurement of atrioventricular valve regurgitant jets using 4D flow cardiovascular magnetic resonance is accurate and reliable for children with congenital heart disease: a retrospective cohort study

**DOI:** 10.1186/s12968-020-00612-4

**Published:** 2020-05-14

**Authors:** Kimberley Jacobs, Joseph Rigdon, Frandics Chan, Joseph Y. Cheng, Marcus T. Alley, Shreyas Vasanawala, Shiraz A. Maskatia

**Affiliations:** 1 Department of Pediatrics, Stanford University School of Medicine, 725 Welch Rd, Room G71, MC 5906, Palo Alto, CA 94304 USA; 2 Department of Medicine, Quantitative Sciences Unit, Stanford University School of Medicine, 300 Pasteur Dr, Palo Alto, CA 94305 USA; 3 Department of Radiology, Divisions of Pediatric Radiology and Cardiovascular Imaging, Stanford University School of Medicine, 300 Pasteur Dr, Palo Alto, CA 94305 USA; 4 Department of Pediatrics, Divisions of Pediatric Cardiology and Cardiovascular Imaging, Stanford University School of Medicine, 300 Pasteur Dr, Palo Alto, CA 94305 USA

**Keywords:** 4DF CMR, 4D flow, Atrioventricular valve regurgitation, Direct measurement of regurgitant jet, Pediatric CHD, MRI

## Abstract

**Background:**

3D-time resolved flow (4DF) cardiovascular magnetic resonance (CMR) with retrospective analysis of atrioventricular valve regurgitation (AVVR) allows for internal validation by multiple direct and indirect methods. Limited data exist on direct measurement of AVVR by 4DF CMR in pediatric congenital heart disease (CHD). We aimed to validate direct measurement of the AVVR jet as accurate and reliable compared to the volumetric method (clinical standard by 2D CMR) and as a superior method of internal validation than the annular inflow method.

**Methods:**

We identified 44 consecutive patients with diverse CHD referred for evaluation of AVVR by CMR. 1.5 T or 3 T scanners, intravenous contrast, and a combination of parallel imaging and compressed sensing were used. Four methods of measuring AVVR volume (RVol) were used: *volumetric method (VOL; the clinical standard)* = stroke volume by 2D balanced steady-state free precession – semilunar valve forward flow (SLFF); *annular inflow method (AIM)* = atrioventricular valve forward flow [AVFF] – semilunar valve net flow (SLNF); and *direct measurement (JET).* AVFF was measured using static and retrospective valve tracking planes. SLFF, SLNF, AVFF, and JET were measured by 4DF phase contrast. Regurgitant fraction was calculated as [RVol/(RVol+SLNF)]× 100. Statistical methods included Spearman, Wilcoxon rank sum test/Student paired t-test, Bland Altman analysis, and intra-class coefficient (ICC), where appropriate.

**Results:**

Regurgitant fraction by JET strongly correlated with the indirect methods (VOL and AIM) (ρ = 0.73–0.80, *p* < 0.001) and was similar to VOL with a median difference (interquartile range) of − 1.5% (− 8.3–7.2%; *p* = 0.624). VOL had weaker correlations with AIM and JET (ρ = 0.69–0.73, *p* < 0.001). AIM underestimated RF by 3.6–6.9% compared to VOL and JET, *p* < 0.03. Intra- and inter- observer reliability were excellent for all methods (ICC 0.94–0.99). The mean (±standard deviation) inter-observer difference for VOL was 2.4% (±5.1%), *p* < 0.05.

**Conclusions:**

In a diverse cohort of pediatric CHD, measurement of AVVR using JET is accurate and reliable to VOL and is a superior method of internal validation compared to AIM. This study supports use of 4DF CMR for measurement of AVVR, obviating need for expert prospective prescription during image acquisition by 2D CMR.

## Introduction

The presence of atrioventricular valve regurgitation (AVVR) causes significant morbidity and mortality in patients with congenital heart disease (CHD) [1–6]. AVVR of moderate or greater degrees is a significant risk factor for unwanted outcomes, including re-operation in patients with repaired atrioventricular septal defects and death or heart transplantation in the single ventricle populations [5–7]. In CHD, the initial mechanism of AVVR is most likely anatomic abnormalities such as septal clefts, dysplasia, or prolapse. As a result of progressive, eccentric regurgitation, a pathologic cycle develops of volume overload, annular dilation, and worsened regurgitation [5, 8]. Although the standard of care for initial evaluation of AVVR is transthoracic echocardiography, accuracy and reliability is limited in distinguishing moderate from severe regurgitation—a distinction particularly important as it impacts surgical management [9–12]. Within this context, cardiovascular magnetic resonance (CMR) has become the clinical standard for calculating AVVR, leveraging its strengths as the gold standard for measuring ventricular volumes and flow [9, 13–17].

The recommended method of measuring AVVR by 2D CMR is through the volumetric (VOL) method—the difference between ventricular stroke volume (by planimetry) and forward flow across the respective semilunar valve (assessed by phase contrast imaging (PC) CMR) [9, 13–18]. Compared to echocardiography, the VOL method has better reliability [9, 10, 16, 19, 20] and accuracy based on correlation with outcome measures including surgical indication and ventricular remodeling after surgery [9, 20, 21]. However, in patients with complex CHD, the reliance on two separate CMR techniques and measurement errors inherent in planimetry can negatively impact accuracy and reliability [16]. The annular inflow method, calculated as the difference between atrioventricular (AV) forward flow and semilunar net flow (both assessed by PC), has similar reliability as the VOL method and can be internally validated by comparison of net inflow and outflow measurements [15, 22–24]. Manual retrospective valve-tracking has improved the accuracy of measuring AV flow by accounting for the basal displacement of the annulus during diastole instead of using a static plane which can overestimate AV flow [23, 25, 26].

In addition to the above two indirect measures of regurgitant flow, direct measurement of the regurgitant jet by a static 2D plane is also possible. This method is limited by dynamic motion of the valve, complex valvular anatomy, number and eccentricity of jets, and change in angulation of the jet during the cardiac cycle [16, 23, 27–29]. Recent advancements in 3D phase-contrast CMR (4D flow or 4DF) including compressed sensing, parallel imaging, and motion correction allow for practical solutions to these limitations with short scan times and creation of manually adjustable planes during post-processing. 4DF is a validated and reliable method for the measurement of flow and volume that has become part of standard clinical imaging at some institutions [22–26, 30–39]. While indirect methods of measuring AVVR have been validated with 4DF CMR [22–24, 36, 37, 40], data on directly measuring AVVR jets remain limited in the pediatric CHD population [24]. Direct measurement of the regurgitant jet has a theoretical advantage in that it is a single measurement unaffected by the aforementioned compounded errors from the indirect methods. We sought to determine the utility of direct measurement of AVVR using 4DF CMR in pediatric patients with CHD compared to currently used indirect measurements. We hypothesized that: (1) direct measurement of the AVVR jet (JET) is accurate and reliable compared to the current clinical standard—the VOL method; and (2) the JET provides a better method of internal validation than the annular inflow method (AIM).

## Material and methods

In this retrospective cohort study, we identified all pediatric patients referred for evaluation of AVVR by CMR with 2D planimetry and 4DF acquisitions at our institution from February 2013 to January 2019. Exclusion criteria included patients with ventricular level shunts, more than one outflow tract from the respective ventricle, and more than trace regurgitation (≥5%) of the respective semilunar valve as measured by 4DF CMR. Although each method of AVVR assessment accounts for semilunar valve regurgitation, there is some variability in that assessment. In order to ensure that any variability identified reflects AV valve regurgitation assessment and not semilunar valve assessment, we excluded patients with significant (mild or worse) regurgitation of the semilunar valve ipsilateral to the regurgitant AV valve. A cohort of 67 patients met inclusion criteria. Of these, seventeen (25%) were excluded due to the presence of mild or greater semilunar valve regurgitation. An additional six (9%) were excluded due to concerns related to image quality including excessive aliasing on 4DF (*n* = 1), movement during 4DF acquisitions (*n* = 2), and lack of visible AVVR jets (*n* = 3). No subjects were excluded due to ventricular level shunt or dual-outflow tract; all subjects had a Qp:Qs ~ 1 except for two subjects with hypoplastic left heart syndrome and a bi-directional Glenn palliation with a Qp:Qs of 0.55–0.77. Forty-four patients comprised the study cohort (Table 1). If a subject had multiple CMR scans during the 6-year inclusion period, the most recent CMR scan was included such that each subject was represented only once in the dataset. For descriptive purposes of this study, in patients with a history of repaired AV canal, the presence of left-sided regurgitation was categorized as mitral regurgitation and right-sided regurgitation was categorized as tricuspid regurgitation. Our local IRB approved this study with waived informed consent.

**Table 1:** Patient Characteristics

	Mitral regurgitation	Tricuspid regurgitation	All	***p-value***
	*(n = 18)*	*(n = 26)*	*(n = 44)*	
Age (years)	12.6 ± 7.8	10.7 ± 5.0	11.4 ± 6.3	*0.370*
Sex (F)	8 (44%)	15 (58%)	23 (52%)	*0.387*
Race				
Caucasian	9 (50%)	8 (31%)	17 (39%)	*0.543*
African American		1 (4%)	1 (2%)	
Hispanic	6 (33%)	11 (42%)	17 (39%)	
Asian	3 (17%)	6 (23%)	9 (20%)	
Height (cm)	141.6 ± 36.3	133.7 ± 28.4	136.9 ± 31.7	*0.420*
Weight (kg)	49.2 ± 32.2	43.2 ± 34.0	45.7 ± 33.0	*0.562*
Aliasing of the jet	12 (67%)	11 (42%)	23 (52%)	*0.112*
Number of regurgitant jets				
1	13 (72%)	21 (81%)	34 (77%)	*0.654*
2	3 (17%)	4 (15%)	7 (16%)	
3	1 (6%)	1 (4%)	2 (5%)	
5	1 (6%)		1 (2%)	
Regurgitation				
Mild (< 30%)	13 (72%)	14 (54%)	27 (61%)	*0.140*
Moderate (31–39%)		3 (11%)	3 (7%)	
Moderate-Severe (40–49%)	1 (6%)	6 (23%)	7 (16%)	
Severe (> 50%)	4 (22%)	3 (11%)	7 (16%)	
Cardiac Condition				
Bacterial endocarditis	2 (11%)		2 (5%)	
ccTGA	1 (6%)	2 (8%)	3 (7%)	
Atrioventricular septal defect	5 (28%)	1 (4%)	6 (14%)	
Dysplastic tricuspid valve		1 (4%)	1 (2%)	
Ebstein anomaly		8 (31%)	8 (18%)	
HLHS s/p Glenn		2 (8%)	2 (5%)	
HLHS s/p Fontan		4 (15%)	4 (9%)	
HOCM	1 (6%)		1 (2%)	
PA/IVS s/p Fontan	1 (6%)		1 (2%)	
Mitral Valve Prolapse	6 (34%)		6 (14%)	
Pulmonary hypertension		7 (27%)	7 (16%)	
Pulmonary stenosis		1 (4%)	1 (2%)	
Shone complex	1 (6%)		1 (2%)	
Supramitral ring	1 (6%)		1 (2%)	
Shunt ratio (Qp:Qs)	1.0 ± 0.08	0.97 ± 0.11	0.98 ± 0.10	*0.956*
Genetic syndromes				
Marfan Syndrome	2 (11%)	1 (4%)	3 (7%)	
Pierre Robin syndrome	1 (6%)		1 (2%)	
Trisomy 21	1 (6%)		1 (2%)	
Turner syndrome		1 (4%)	1 (2%)	
Field Strength				
1.5 T	8 (44%)	17 (65%)	25 (57%)	*0.168*
3 T	10 (56%)	9 (35%)	19 (43%)	
Contrast				
Gadobenate	5 (27%)	11 (42%)	16 (36%)	*0.200*
Gadofosveset	1 (6%)	4 (15%)	5 (11%)	
Ferumoxytol	9 (50%)	11 (42%)	20 (45%)	
No contrast	1 (6%)		1 (2%)	

*p*-values refer to comparisons between mitral and tricuspid regurgitation groups. Severity of regurgitation is classified based on VOL measurements. Continuous variables compared with Student’s t-test, categorical variables compared with Pearson’s chi-square test. *F* indicates female, *cm* centimeters, *kg* kilograms, *BSA* body surface area, *m*^*2*^ meters squared, *ccTGA* congenitally corrected transposition of the great arteries, *VOL* volumetric method

### Image acquisition

The magnetic field, intravenous contrast, and technical parameters were prescribed by radiologists with expertise in CMR as per routine clinical practice. CMR exams were performed on 1.5 T CMR system (General Electric Healthcare, Waukesha, Wisconsin, USA; Optima 450 W) and 3 T (General Electric Healthcare, GE MR750) scanners. At our institution, it is standard practice with 4DF CMR to use ferumoxytol (Feraheme; AMAG Pharmaceuticals, Waltham, Massachusetts, USA) for 3 T and gadolinium-based contrasts (Gadobenate dimeglumine [Multihance, Bracco Diagnostics, Monroe, New Jersey, USA]; Gadofosveset) for 1.5 T scans to optimize signal-to-noise ratio [41].

Two-dimensional CMR planimetry utilized short-axis, balanced steady-state free-precession (bSSFP) and PC sequences with retrospective gating. Short-axis slices were obtained with a breath hold using one signal average and parallel imaging acceleration; 12–14, 8 mm slices spanned the ventricles at 20 phases per cardiac cycle. Matrix and temporal resolution parameters were adjusted to accommodate breath holding capacity. In patients who could not breath hold, multiple signal averages were employed. 2D PC measurements of atrioventricular forward flow (AVFF) were obtained with a velocity encoding (VENC) of 150 m/s as default, then increased until no aliasing was noted. The following parameters were used: temporal resolution 36.4 msec (range 21–54), acquired spatial resolution 256 × 192, and slice thickness 10 mm. There was no adjustment for through-plane motion for 2D PC measurements of AVFF. 2D PC measurements of AVFF were available for only 13/44 (30%) patients, thus 4DF CMR AVFF measurements via a static plane were obtained as a substitute for the purposes of this study.

4DF acquisitions were obtained as the last sequence of each CMR study following administration of contrast in the majority of patients (Table 1). Non-contrast enhanced 4DF acquisition was obtained in one subject with a historical allergy to gadolinium-based contrast. 1.5 T CMR images were obtained with the following parameters, mean (range): flip angle of 15*, reconstructed spatial resolution of 1.2 (0.8–1.5) × 1.4 (0.8–1.8) × 2.1 (0.9–3) mm^3^, echo time 1.9 (1.5–2.1) msec, repetition time 4.4 (3.6–5.1) msec, views-per-segment of 3 (2–5) depending on heart rate, temporal resolution of 62.5 msec (28.6–83.6), bandwidth of ±63.3 kHz (62–125), and VENC parameters of 300 (250–450 m/sec) chosen to avoid aliasing. The base acceleration factor used was 5 × 3 with motion correction performed by leveraging the velocity encoding gradients as intrinsic navigators and soft-gating the data, for an approximate overall acceleration factor of 30. 3 T CMR images were obtained with the following parameters, mean (range): flip angle of 15*, reconstructed spatial resolution of 0.84 (0.7–1.2) × 0.9 (0.7–1.6) × 1.7 (1.2–2.8) mm^3^, TE 1.8 (1.7–2.1) msec, TR 4.1 (3.7–4.8) msec, true temporal resolution of 32.4 msec (18.3–58.6), bandwidth of ±83.3 kHz (63–100), total acceleration factor of 2.0 × 2.0, and VENC 300 (250–550 m/sec) chosen to avoid aliasing. Retrospective gating was used to obtain a total of 20 cardiac phases for 1.5 T and 20–30 phases for 3 T scanners. Due to clinical workflow, many of our 3 T exams are performed sedated or with general anesthesia and the vast majority of the exams performed on the 1.5 T magnet are performed unsedated. For this reason, there is a greater effort to accelerate sequences performed on our 1.5 T magnet.

To minimize coherent artifacts from respiratory motion, a pseudo-random view-ordering and sampling was used for 4DF [42]. More specifically, a variable-density Poisson disc sampling mask was first generated [43]. Each (ky,kz)-sample was then grouped into radial spokes and ordered according to the golden-ratio [44]. Image reconstruction was performed with a combined parallel-imaging compressed sensing algorithm, with soft-gating of the source data based on intrinsic respiration motion navigation [42, 43, 45]. Temporal and spatial sparsity were enforced. Post-processing software corrected for phase errors by estimating the background phase from a polynomial fit on the phase of the static tissue.

### Image analysis

Subjects were analyzed in a blinded fashion by KGJ, a pediatrics resident with 2 years research experience in CMR post-processing. For training, a series of 10 subjects were blindly measured with feedback from a cardiologist with > 8 years clinical experience in CMR (SAM). The data from these feedback sessions were not included in the study. Subjects were placed in a random order by a random number generator for analysis such that the effect of training would be minimized. A random number generator was used to select 20 subjects for which measurements were repeated with a minimum of 2 weeks between analyses to obtain intra- and inter-rater reliability (KGJ, SAM).

2D planimetric images were analyzed on QMassMR (MEDIS; Leiden, the Netherlands). A parallel stack of short-axis images were manually traced for endocardial borders to calculate a volume for each image plane, which were then summed to produce end-diastolic volumes (EDV) and end-systolic volumes (ESV). Both the 2- and 4-chamber views were used to cross-reference for ventricular planimetry [16]. A slice was considered to contain primarily atrium when < 50% of the blood volume was encircled with myocardium, outflow tracts were included in ventricular volumes up to the semilunar valve cusps, and trabeculations and papillary muscles were considered part of the blood pool [14]. Stroke volume (SV) was calculated as EDV-ESV for each respective ventricle. 2D PC measurements of AVFF were analyzed on QFlowMR (MEDIS). A region of interest was traced around the AV valve with the velocity therein integrated to determine the flow volume [15]. A sub-group analysis was performed between 2D PC AVFF and 4DF static AVFF. However, for homogeneity across the study cohort, all calculations of AIM_stat_ utilized the 4DF CMR measurement of AVFF.

4DF analyses were performed using cloud-based software (Arterys, San Francisco, California, USA). Measurements of aortic and pulmonary artery flow were made at the level of the sinotubular junction for the aorta and midway between the pulmonary valve and pulmonary artery bifurcation, respectively, as per published recommendations [14, 28]. With reference to the 2- and 4-chamber cross-sections, an initial plane was created at end-systole at the mid-leaflet level of the AV valve and orthogonal to flow [16, 27]. Flow was visualized by velocity vectors depicting blood flow directionality in real-time and color-coded for magnitude. Flow through the AV static plane was semi-automatically traced by the software during diastole and manually corrected, as needed. The retrospective valve-tracking AV plane was manually adjusted through diastole to remain near the mid-leaflet level and orthogonal to flow [37], with similar semi-automated tracing of the AV inflow as with the static plane. The regurgitant jet was measured by a plane perpendicular to the jet immediately distal to regions of aliasing, if present [24, 40]. In cases of multiple jets, separate planes were created for each jet and the volume of each jet summed. Care was taken to ensure tracings for each individual jet did not overlap. Time necessary to complete post-processing was recorded for both AIMs and JET.

**Fig. 1:**
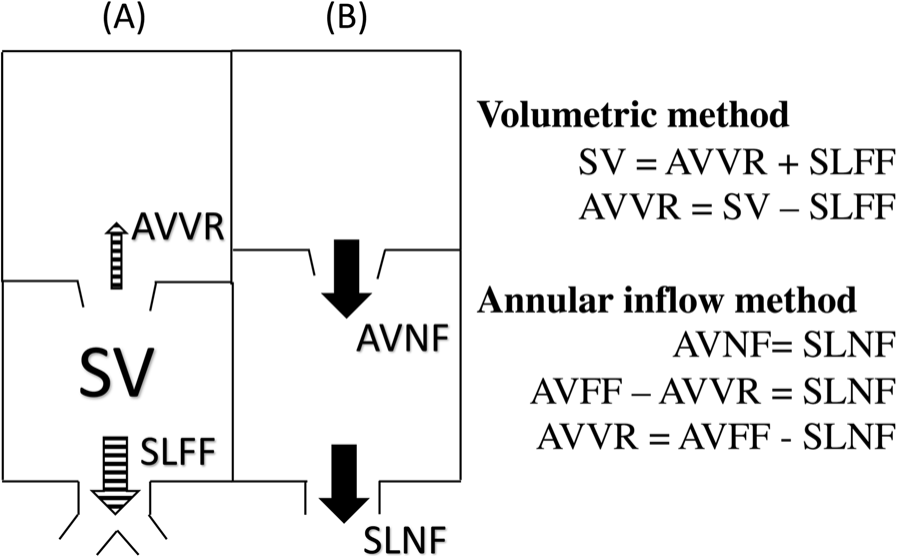
Graphic and mathematical depiction of the indirect methods calculating atrioventricular valve regurgitation (AVVR). **a** Ventricular stroke volume (SV) is the sum of AVVR and semilunar forward flow (SLFF); **b** AVVR is derived from the conservation of mass which assumes that atrioventricular net flow (AVNF) equals semilunar net flow (SLNF) in the absence of an intracardiac shunt

**Fig. 2:**
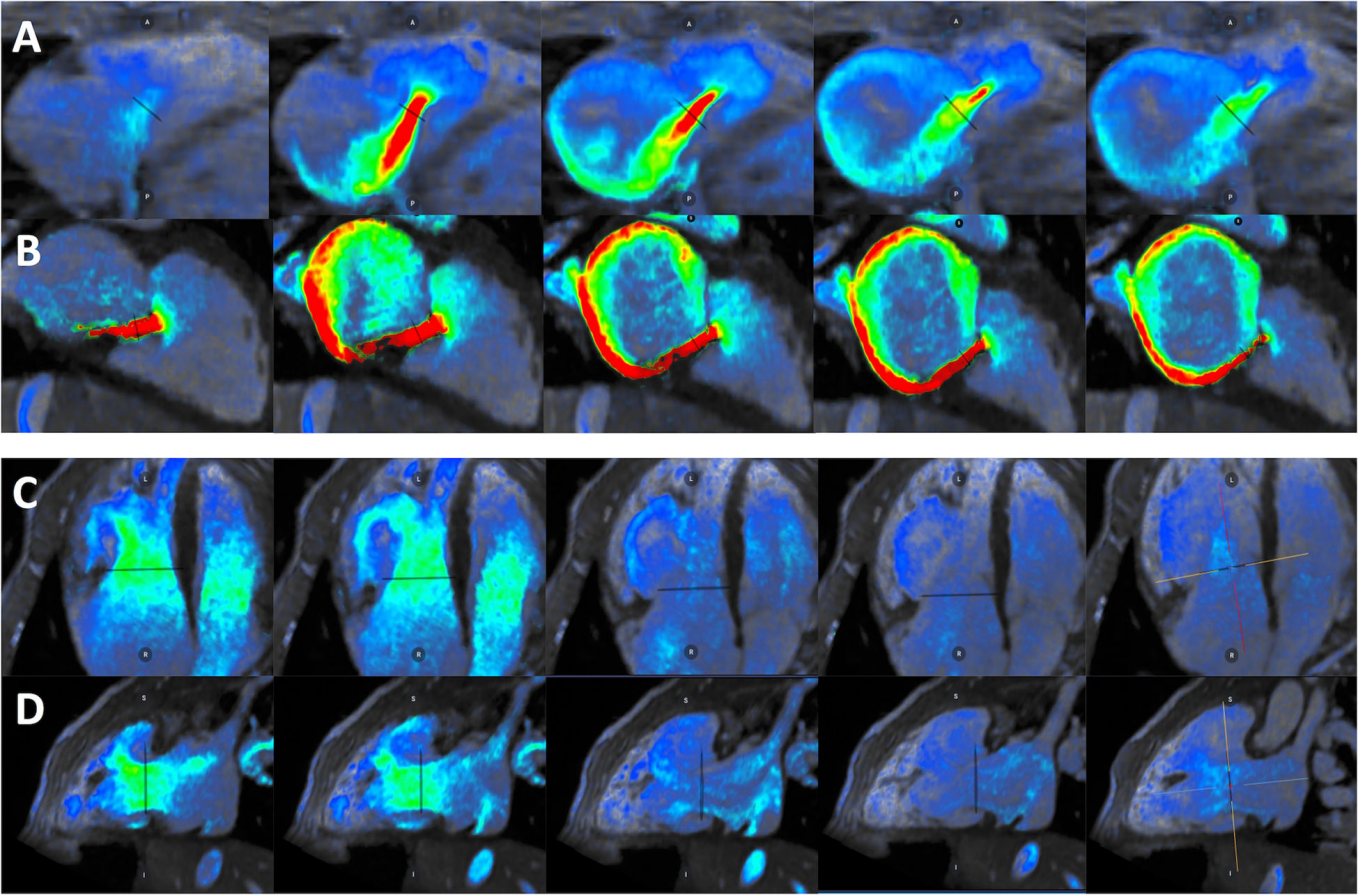
4DF CMR on Arterys of the direct jet method (**a**, **b**) and the indirect annular inflow method (AIM_track_) by valve tracking planes (**c**, **d**). Examples of tricuspid regurgitation (row **a**) and mitral regurgitation (row **b**) are presented. Rows (**c**) and (**d**) represent four-chamber and two-chamber views of AIM_track_

Our study compares four methodologies calculating regurgitant volume (RVol). Given the clinical utility of regurgitant fraction (RF), we report RF as our outcome. RF was calculated as the quotient of AVVR and AV inflow by the formula *RVol/[RVol + semilunar valve net flow (SLNF)]× 100* based on conservation of mass dictating that net inlet and outlet flows are equal. RF was classified as mild (< 30%), moderate (30–39%), moderate-severe (40–49%), or severe (> 50%) [13]. The four methods of calculating RVol were as follows (Figs. 1 and 2; Supplementary videos):

1. Volumetric method (VOL) = stroke volume by 2D bSSFP – forward flow across the semilunar valve by 4DF PC
2. Annular inflow method (AIM_stat_) = AVFF, using a static plane, by 4DF PC – SLNF by 4DF PC
3. Annular inflow method (AIM_track_) = AVFF, using a valve-tracking plane, by 4DF PC – SLNF by 4DF PC
4. Direct measurement of the AVVR jet (JET) by 4DF PC

### Statistical analysis

A power analysis demonstrated that for a paired t-test, 44 subjects would be necessary with alpha of 0.05 and beta of 10% (power 90%) to detect a 10% difference in regurgitant volume between subjects, allowing for 20% variance (effect size: 0.5).

Continuous data were described as mean (±standard deviation (SD)) and median (interquartile range (IQR)) as appropriate based on normality assessment with Shapiro-Wilk tests and histograms. Correlation between modalities was determined via Pearson’s (r) or Spearman’s (ρ) correlation coefficients, as appropriate. Variance between measurements was described as median difference (IQR) and absolute median difference (IQR) or mean difference (±SD) and absolute mean difference (±SD), as appropriate. The presence of any systematic difference between measurements was determined via the Wilcoxon signed rank test or Student’s paired t-test. All differences were calculated in the order presented, e.g. VOL vs. AIM calculated as VOL - AIM. Bland-Altman plots were used to visualize and compare agreement between methods [46, 47]. Bland-Altman plots show variance as limits of agreement (LOA), calculated as mean ± SDx1.96. However, this does not appropriately represent our data given the skewed, non-normal distribution, thus consideration should be given to the median difference (IQR) provided in the table as additional representation of the distribution of data. Linear regression scatter plots were used to further visualize distribution and correlation of data. Spearman’s correlation coefficients are presented on the linear regression analyses as the non-parametric complement.

Reliability was assessed with intra- and inter-rater repeated measurements (KGJ, SAM) [48]. Intra-class correlation coefficients (ICC) were calculated using a two-way mixed model with absolute agreement, reporting the single measures ICC with 95% confidence interval (CI) [49]. Statistical significance was considered if a two tailed *p*-value was < 0.05. All statistical analyses were performed using SPSS (v.25, Statistical Package for the Social Sciences, International Business Machines, Inc., Armonk, New York, USA).

## Results

Forty-four pediatric patients (11.4 ± 6.3 years; 23 (52%) female) with diverse CHD diagnoses were included in this study (Table 1). Eighteen (41%) had mitral regurgitation. To tolerate CMR, seven (16%) subjects underwent CMR with general anesthesia and four (9%) had conscious sedation. Five additional subjects (11%) were sedated due to simultaneous procedures or were already sedated prior to CMR. Demographic characteristics are further described in Table 1. We found no significant differences in these characteristics between the populations with mitral and tricuspid regurgitation. The majority of subjects (77%) had one regurgitant jet. Aliasing within the regurgitant jet was common (23/44, 52%) for both mitral and tricuspid regurgitation (*p* = 0.112). Regurgitant fractions ranged from 0.5 to 79% as measured by VOL. The subject with 79% RF was measured similarly by the AIM methods (82–83%). This subject passed away shortly after the exam. The majority of subjects had mild RF; however, five subjects by VOL, six by AIM_stat_, and seven by AIM_track_ were calculated to have negative regurgitant fractions (Table 2). Heart rates were similar between 2D and 4DF scans: 80.0 (± 19.7) bpm for 2D bSSFP (*n* = 44) vs. 85.2 (± 18.4) bpm for 4DF CMR, *p* = 0.308; 86.6 (± 21.6) bpm for 2D PC AVFF (*n* = 13) vs. 89.5 (± 20.0) bpm for 4DF CMR, *p* = 0.253. Post-processing times for AVVR using 4DF CMR were acceptable for clinical practice: AIM_stat_ 2.8 ± 0.7 min, AIM_track_3.7 ± 1.9 min, and JET 3.1 ± 2.1 min (*p* = 0.114). 4DF acquisition time was 8.4 (7.6–9.7) and 14.7 (12.1–15.2) (*p* = 0.001) minutes for 1.5 T and 3 T studies, respectively.

**Table 2:** Comparison of the RF as measured by each method

	Median (IQR)	Mild (0–30%)	Moderate (31–39%)	Moderate - Severe (40–49%)	Severe (>49 %)
**VOL**	21.4% (7.5–42.3%)	22^a^	3	7	7
**AIM**_**stat**_	19.2% (5.8–30.0%)	27^a^	2	4	5
**AIM**_**track**_	16.4% (4.5–27.4%)	27^a^	3	3	4
**JET**	22.1% (9.9–37.6%)	27	9	3	5

^a^5 subjects by VOL, 6 by AIM_stat_, and 7 by AIM_track_ had negative regurgitant fractions and were not included in this table. *VOL* Volumetry method, *AIM*_*stat*_ Annular inflow method by static plane, *AIM*_*track*_ Annular inflow method by valve-tracking plane, *JET* Direct measurement of the atrioventricular regurgitant jet

### Comparison between methods to assess regurgitant fraction

Measurement of RF by JET was similar to VOL (median difference (IQR) -1.5% (− 8.3–7.2%), *p* = 0.62) with moderate-strong correlation (ρ = 0.73, *p* < 0.001) (Table 3). While there were moderate-strong correlations between VOL and both AIM methods (ρ = 0.69–0.70, p < 0.001), the AIM methods using static (AIM_stat_) and valve-tracking planes (AIM_track_) were significantly different from VOL and underestimated RF with median differences of 4.3% [− 2.0–15.7%] (*p* = 0.027) and 6.9% [0.2–15.0%] (*p* = 0.006), respectively. These methods similarly underestimated RF relative to JET, though to a slightly lesser degree, with median differences of 3.6% [− 12.0–1.8%] (*p* = 0.005) and 5.8% [− 13.7–0.3%] (*p* < 0.001), respectively. AIM_stat_ and AIM_track_ were highly correlated (ρ = 0.98, *p* < 0.001). RF by AIM_stat_ was slightly larger than AIM_track_ by a median difference (IQR) of 1.7% [0.1–4.3%] (*p* < 0.001).

**Table 3:** Comparison of regurgitant fraction (RF) measured by the indirect and direct methods

	Spearman (ρ)	WSRT	Median difference (IQR)	Absolute Median difference (IQR)
**VOL vs. AIM**_**stat**_	ρ = 0.69	*p* = 0.027	4.34% (− 2.02–15.73)	8.97% (4.07–21.94)
**VOL vs. AIM**_**track**_	ρ = 0.70	*p *= 0.006	6.87% (0.24–14.97)	11.70% (4.80–23.69)
**VOL vs. JET**	ρ = 0.73	*p* = 0.624	−1.49% (− 8.34–7.21)	8.24% (3.90–14.98)
**AIM**_**stat**_**vs. AIM**_**track**_	ρ = 0.98	*p* < 0.001	1.67% (0.12–4.27)	1.88% (1.19–4.27)
**AIM**_**stat**_**vs. JET**	ρ = 0.80	*p* = 0.005	−3.57% (− 12.03–1.75)	7.52% (2.25–13.45)
**AIM**_**track**_**vs. JET**	ρ = 0.80	*p* < 0.001	−5.80% (− 13.65 – − 0.32)	7.40% (3.11–13.65)

Differences are calculated in the order presented (ex: VOL vs. JET = VOL-JET). *VOL* Volumetry method, *AIM*_*stat*_ Annular inflow method by static plane, *AIM*_*track*_ Annular inflow method by valve-tracking plane, *JET* Direct measurement of the atrioventricular regurgitant jet, *IQR* Interquartile range, *WSRT* Wilcoxon signed rank test

**Fig. 3:**
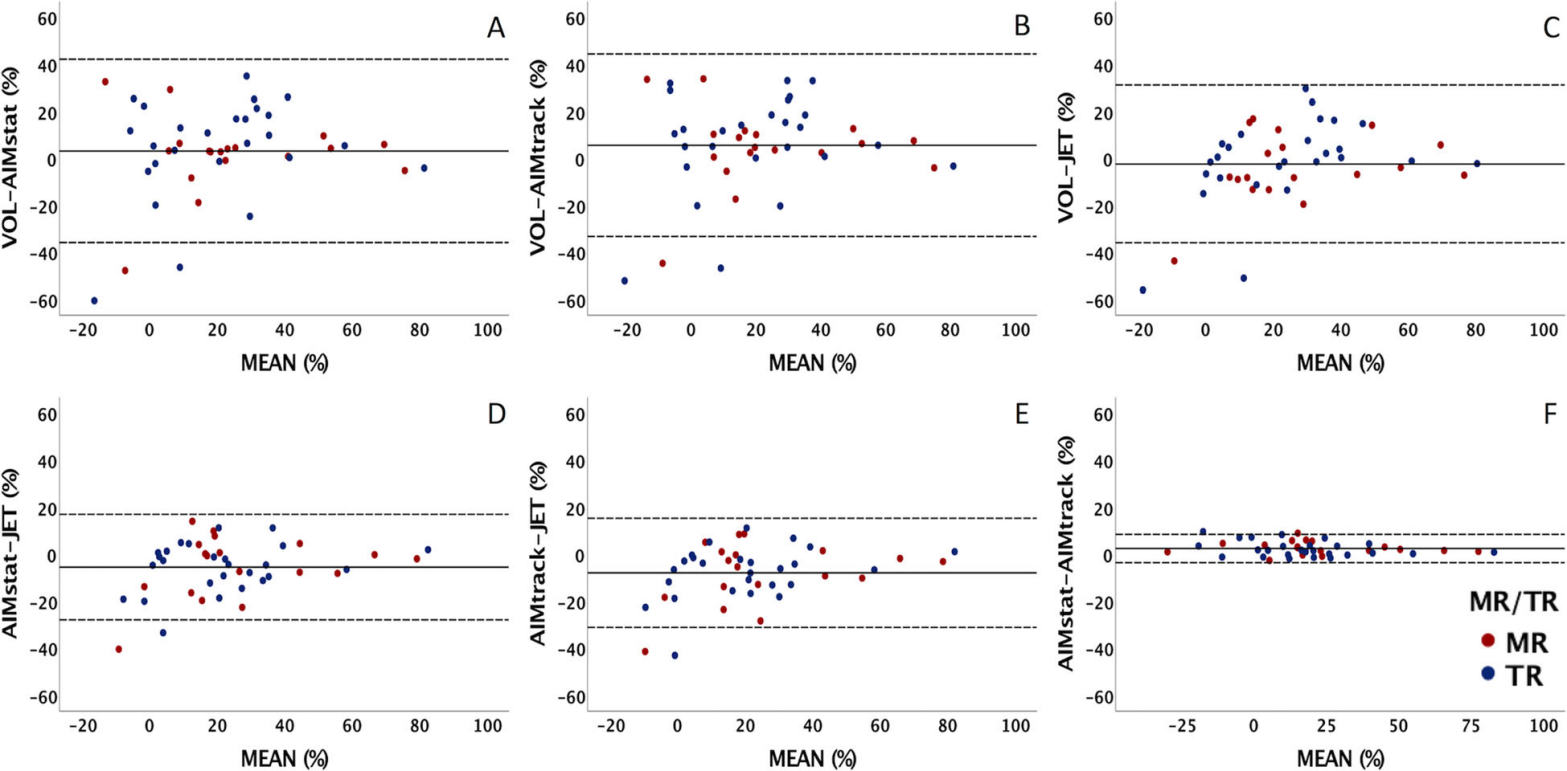
Bland Altman plots comparing the 4 methods in this study: the indirect methods (VOL and both AIMs) and the direct method (JET). The limits of agreements (dashed lines) are narrower for the JET comparisons (**c**-**e**) than for the VOL comparisons (**a**-**c**). The mean difference is shown as a solid line. Mitral regurgitation (MR) is denoted by red points, tricuspid regurgitation (TR) is denoted by blue points. VOL = volumetry method, AIM_stat_ = annular inflow method by static plane, AIM_track_ = annular inflow method by valve-tracking plane, JET = direct measurement of jet

**Fig. 4:**
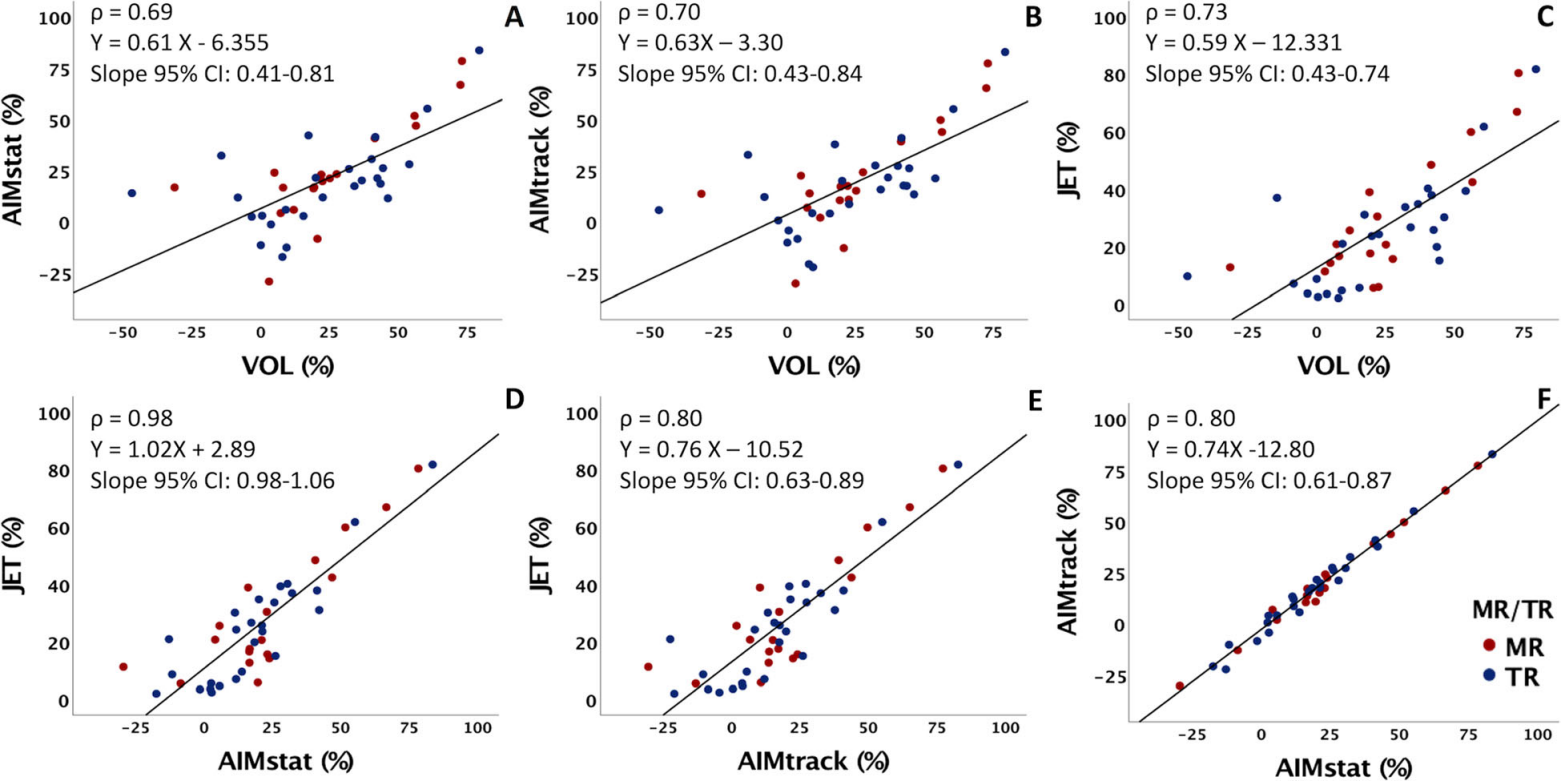
Linear regression plots comparing the 4 methods of this study: the indirect methods (VOL and both AIMs) and the direct method (JET). Given non-parametric distribution of data, Spearman’s correlation coefficients are substituted for Pearson’s. Mitral regurgitation (MR) is denoted by red points, tricuspid regurgitation (TR) is denoted by blue points. VOL = volumetry method, AIM_stat_ = annular inflow method by static plane, AIM_track_ = annular inflow method by valve-tracking plane, JET = direct measurement of jet

In assessing internal consistency, BA and scatter plots comparing all four methods demonstrated narrower limits of agreements and more precise spread of data for the JET method compared to VOL and AIM methods than the VOL method compared to JET and AIM methods (Figs. 3 and 4).

For the 13 subjects in whom 2D PC AVFF data was available, a sub-group analysis was performed. AVFF measurements were not significantly different between 2D PC and 4DF CMR (mean difference 0.4 ± 9.5 mL/beat, *p* = 0.892) and were highly correlated (Pearson’s r = 0.962, *p* < 0.001). AIM_stat_ using 2D PC AVFF calculated a lower RF than AIM_stat_ using 4DF static AVFF though this difference was non-significant (median difference (IQR) 2.8% (− 8.1–5.5), *p* = 0.701)). As a result, in this smaller cohort, 2D AIM_stat_ correlated worse with VOL (ρ =0.736, *p* = 0.004) than 4DF AIM_stat_ with VOL (ρ =0.824, *p* = 0.001). AIM_stat_ by both modalities underestimated RF relative to VOL by median differences of 9.1–11.7% (*p* < 0.05).

### Reliability

**Fig. 5:**
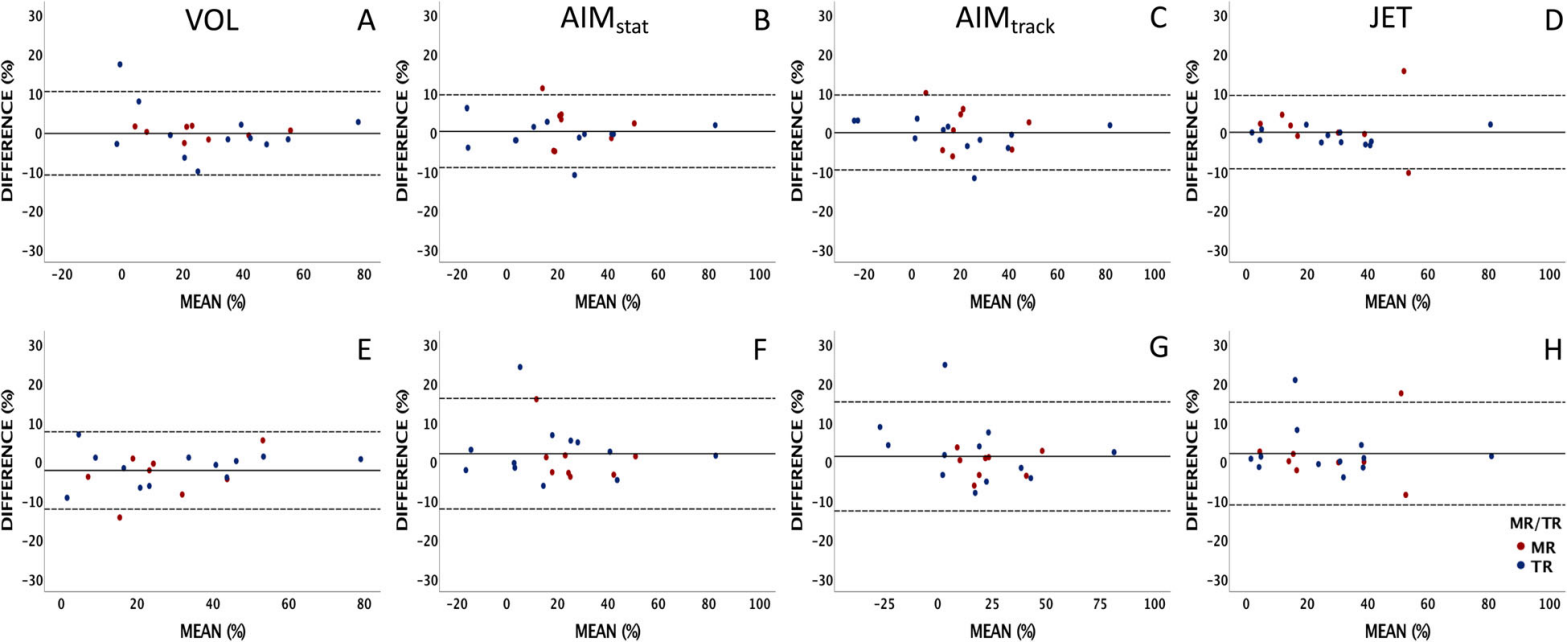
Bland Altman plots comparing the intra- and inter-observer reliability measurements (**a**-**d** and **e**-**h**, respectively) using the indirect and direct methods measuring atrioventricular valve regurgitation. Mean difference is represented with a solid line; limits of agreement are represented with dashed lines. Mitral regurgitation (MR) is denoted by red points, tricuspid regurgitation (TR) is denoted by blue points. VOL = volumetry method, AIM_stat_ = annular inflow method by static plane, AIM_track_ = annular inflow method by valve-tracking plane, JET = direct measurement of jet

Intra- and inter-observer reliability was tested on 20 randomly selected subjects (45%): 12 had tricuspid regurgitation and 8 had mitral regurgitation. Intra-observer correlations for the four methods were excellent (ICC 0.97–0.98) with no significant differences between measurements (*p* = 0.88–0.97) (Table 4). Mean differences were all close to zero with narrow LOAs (Fig. 5). Inter-observer correlations for the 4 methods were also excellent (ICC 0.94–0.96). Measurements of RF were not significantly different between raters using the AIM and JET methods (*p* = 0.22–0.47), with small mean differences and narrow LOAs. However, inter-observer measurements of VOL were significantly different (*p* = 0.046), although the clinical significance of this difference was small (2.41% ± 5.05) and the variance of this difference was smaller than inter-observer differences for JET and AIM methods (Fig. 5). Intra- and inter-observer analysis of planimetry measurements demonstrated excellent reliability of left ventricular (LV) and right ventricular (RV) volumes (ICC = 0.99) with small, clinically insignificant, median differences (Table 4).

**Table 4:** Intra- and inter-observer reliability

		Intra-observer		Inter-observer	
**Regurgitant Fraction (%)** *(n = 20)*		**ICC (95% CI)**	**Mean Difference (± SD)**	**ICC (95% CI)**	**Mean Difference (± SD)**
VOL	28.3% ± 20.6	0.97 (0.92–0.99)	− 0.04% ± 5.43	0.96 (0.90–0.99)	− 2.41% ± 5.05*
AIM_stat_	23.3% ± 22.5	0.98 (0.95–0.99)	0.15% ± 4.72	0.95 (0.88–0.98)	1.89% ± 7.23
AIM_track_	20.4% ± 23.4	0.98 (0.95–0.99)	− 0.17% ± 4.86	0.96 (0.90–0.98)	1.18% ± 7.12
JET	28.6% ± 20.1	0.97 (0.93–0.99)	− 0.09% ± 4.77	0.94 (0.87–0.98)	1.92% ± 6.71
**Planimetry** *(LV = 8; RV = 12)*		**ICC (95% CI)**	**Median Difference (IQR)**	**ICC (95% CI)**	**Median Difference (IQR)**
LVEDVi (mL/m^2^)	107.8 (84.5–128.7)	0.99 (0.97–0.99)	− 0.9 (− 4.3–0.4)	0.99 (0.96–0.99)	− 1.6 (− 4.3–2.1)
LVESVi (mL/m^2^)	52.5 (40.3–59.3)	0.99 (0.95–0.99)	0.3 (− 5.1–1.7)	0.99 (0.96–0.99)	1.4 (0.3–3.6)
RVEDVi (mL/m^2^)	135.6 (121.0–214.3)	0.99 (0.99–1.00)	− 1.8 (− 6.9–2.1)	0.99 (0.99–1.00)	− 0.5 (− 2.3–2.4)
RVESVi (mL/m^2^)	74.4 (67.5–140.2)	0.99 (0.98–0.99)	− 2.6 (− 5.0–2.7)	0.99 (0.99–0.99)	1.4 (− 0.1–4.7)**

Descriptive statistics for the 20 subjects from the original dataset are depicted in the second column to which the intra- and inter-observer differences can be compared. These differences were non-significant (*p* > 0.1), except for VOL and RVESVi inter-observer analysis as denoted by asterisks (*p = 0.046 and **p = 0.033, respectively). *VOL* Volumetry method, *AIM*_*stat*_ Annular inflow method by static plane, *AIM*_*track*_ Annular inflow method by valve-tracking plane, *JET* Direct measurement of the atrioventricular regurgitant jet, *LV* Left ventricle, *RV* Right ventricle, *EDVi* Indexed end-diastolic volume, *ESVi* Indexed end-systolic volume, *ICC* Intra-class correlation, *CI* Confidence interval, *SD* Standard deviation

### Mitral vs tricuspid regurgitation

A sub-group analysis found that agreement between VOL and JET measurements was not affected by laterality of AVVR (Table 5). However, JET had better precision with VOL and AIM methods for assessment of tricuspid RF (ρ = 0.66–0.85, *p* < 0.001; lower measurement variability) than VOL with JET and AIM methods (ρ = 0.58–0.66, *p* < 0.001). Conversely, the JET method was less precise with VOL and AIM for assessment of mitral RF (ρ = 0.66–0.69, p < 0.001; higher measurement variability) than VOL with JET and AIM (ρ = 0.66–0.79, p < 0.001) (Table 5). Measurements of intra- and inter-observer reliability were similar for mitral and tricuspid AVVR.

**Table 5:** Comparison of indirect and direct methods by laterality of AVVR

	Mitral regurgitation			Tricuspid regurgitation		
	**Spearman**	**WSRT**	**Median Difference** **(IQR)**	**Spearman**	**WSRT**	**Median Difference** **(IQR)**
VOL vs. AIM_stat_	ρ = 0.79	*p* = 0.231	3.1% (− 2.0–6.0)	ρ = 0.58	*p* = 0.078	8.1% (−2.7–19.0)
VOL vs. AIM_track_	ρ = 0.76	*p* = 0.078	5.5% (− 0.6–10.6)	ρ = 0.61	*p* = 0.036*	11.1% (− 0.7–20.0)
VOL vs. JET	ρ = 0.66	*p* = 0.349	−7.0% (− 10.1–7.3)	ρ = 0.66	*p* = 0.869	−0.4% (− 7.1–8.2)
AIM_stat_ vs. AIM_track_	ρ = 0.98	*p* = 0.001*	2.4% (1.1–4.8)	ρ = 0.98	*p* = 0.004*	1.6% (−0.3–3.8)
AIM_stat_ vs. JET	ρ = 0.66	*p* = 0.157	−1.3% (− 14.5–4.2)	ρ = 0.85	*p* = 0.014*	−4.5% (− 11.5–1.4)
AIM_track_ vs. JET	ρ = 0.69	*p* = 0.018*	−4.3% (− 15.0–1.1)	ρ = 0.85	*p* = 0.001*	−6.4% (− 13.8 – − 1.2)
	**ICC**	**Paired T-Test**	**Mean Difference** **(± SD)**	**ICC**	**Paired T-Test**	**Mean Difference** **(± SD)**
**Intra-observer reliability**						
VOL	0.996	*p* = 0.844	−0.12% ± 1.64	0.962	*p* = 0.995	0.01% ± 7.01
AIM_stat_	0.918	*p* = 0.397	1.71% ± 5.37	0.989	*p* = 0.472	−0.89% ± 4.23
AIM_track_	0.931	*p* = 0.652	0.96% ± 5.76	0.990	*p* = 0.467	−0.93% ± 4.27
JET	0.935	*p* = 0.591	1.42% ± 7.18	0.995	*p* = 0.074	−1.10% ± 1.92
**Inter-observer reliability**						
VOL	0.909	*p* = 0.133	−3.60% ± 5.98	0.981	*p* = 0.232	−1.62% ± 4.42
AIM_stat_	0.902	*p* = 0.751	0.76% ± 6.51	0.959	*p* = 0.269	2.64% ± 7.85
AIM_track_	0.973	*p* = 0.586	−0.70% ± 3.45	0.957	*p* = 0.353	2.44% ± 8.70
JET	0.928	*p* = 0.644	1.25% ± 7.35	0.954	*p* = 0.238	2.36% ± 6.54

An asterisk (*) denotes statistical significance defined as *p* < 0.05. *VOL* Volumetry method, *AIM*_*stat*_ Annular inflow method by static plane, *AIM*_*track*_ Annular inflow method by valve-tracking plane, *JET* Direct measurement of the atrioventricular regurgitant jet, *ICC* Intra-class correlation, *SD* Standard deviation, *IQR* Interquartile range, *WSRT* Wilcoxon signed rank test

## Discussion

We performed a retrospective study designed to compare the accuracy and reliability of directly measuring AVVR in pediatric CHD with 4DF CMR compared to the standard indirect methods. There were three major findings of our study. (1) Our data demonstrate that assessment of AVVR by JET was overall the most precise measurement, particularly in subjects with tricuspid regurgitation. Though all four methods performed well, the JET correlated with VOL better than AIM and JET correlated with AIM better than VOL. Unlike AIM, the JET method was found to be accurate compared to VOL -- the clinical standard. Further, the JET method showed excellent inter- and intra-observer reliability in a diverse population of pediatric CHD, unaffected by the presence of aliasing or laterality of AVVR. (2) VOL had weaker correlations and larger measurement variability compared with the JET and AIM methods than JET vs. VOL and AIM methods. Though intra- and inter-observer reliability of VOL by ICC were excellent, inter-observer reliability measurements showed a small but statistically significant difference between observers. (3) Despite strong correlations, the AIM using static and valve-tracking techniques underestimated AVVR relative to the JET and VOL methods. Though the degree of this underestimation was small in most subjects, these findings support JET as an alternative measure of internal validation in measuring AVVR.

### Measurement of AVVR

This is the first study demonstrating validity of the JET method to accurately and reliably measure AVVR compared to multiple indirect methods in a diverse pediatric cohort with CHD. Our findings support the results of prior work on the JET method in other patient populations. A retrospective study of 21 adults without CHD found that RF directly measured by 4DF CMR was highly reliable and accurate compared to the VOL method [40]. The correlation and measurement variability noted between the JET and VOL methods in our cohort are similar to prior data from pediatric and adult cohorts [24, 29].

Although this is the first such report in children, the JET method by 4DF CMR has been previously found in adults to have high accuracy and reliability relative to the VOL method, particularly in measuring tricuspid regurgitation [40]. 4DF CMR post-processing allows for visualization of flow to aid in the positioning of measurement planes through the use of velocity vectors [37]. Prior work has demonstrated improved accuracy for the AIM method when the atrioventricular plane is placed at the peak inflow velocity as opposed to the valvular plane [37, 50]. The importance of a measurement plane perpendicular to flow likely extends to JET planes and may explain the relatively worse correlation between measurements of mitral RF between the JET and the VOL and AIM methods. Tricuspid regurgitant jets tend to be more central and laminar in orientation as opposed to mitral jets which are more commonly eccentric with a large angle change during systole [40]. In a cohort of 32 children and adults with repaired atrioventricular septal defect (AVSD) and left-sided regurgitation, the regurgitant jets were found to be non-circular, dynamic, and eccentric with a median angulation change of 30–36° during systole [24]. These features increase the technical difficulty of maintaining an orthogonal plane to the mitral regurgitant jet through systole. As can be seen in a subject with repaired AVSD and resultant mitral cleft (Fig. 2b), the large eccentric regurgitant jet which projects along the inferior mural aspect of the left atrium throughout systole complicates creating a true cross-sectional plane. Five of the 18 (28%) subjects in our cohort with mitral regurgitation had a history of AVSD which may have affected accuracy of our JET measurements despite excellent reliability. Our data highlight the clinical applicability of the JET method in pediatric CHD requiring evaluation of tricuspid regurgitation. Although the results were also promising in cases of mitral regurgitation, considerably eccentric or dynamic jets may affect the accuracy of the JET method.

Our initial intent was to use VOL as the clinical standard to which to compare JET and AIM methods. Upon analysis of the data we found that all four methods were fairly consistent, but that VOL was not the most precise measurement -- which raises the question of its role as the clinical standard. The accuracy of VOL is affected by measurement errors inherent in planimetry, particularly given the practice variation in basal slice inclusion and contour selection, which may have contributed to lower measurement precision [16, 51]. However, our planimetry reliability analyses found excellent intra- and inter-observer reliability for both LV and RV measurements. Similarly, our results demonstrated excellent reliability for the VOL method, supporting the results from multiple prior works [9, 17, 29, 40]. Two additional practical concerns with the VOL method arose from our data. In our cohort, VOL classified more subjects with moderate or severe regurgitation than the JET and AIM methods. This may result in greater consideration for surgery when VOL is used to measure regurgitation. Furthermore, a limitation to the use of VOL as the clinical standard are cases in which the indirect calculation results in a negative RF (Table 2). Negative RFs were found in 5/44 (11%) subjects by the VOL method as well as in a separate 6/44 (14%) and 7/44 (16%) by AIM_stat_ and AIM _track_, respectively.

The AIM methods systematically underestimated RF compared to VOL and JET methods. Though these differences may be clinically insignificant in certain patients, for subjects with mild regurgitation (~ 60% of our cohort), these differences represented up to 25% of their total RF. AIM_stat_ measured slightly but consistently larger than AIM_track_, consistent with prior studies demonstrating that static AV planes overestimate RVol and RF relative to tracking planes [23, 37, 52]. The differences found in our study were smaller than prior reports and may be due to our methodology; determination of a “static plane” by 4DF CMR has significantly more flexibility than a standard 2D CMR scan and may have increased accuracy of the measurements. This is supported by our sub-group analysis demonstrating that AIM_stat_ calculated using 2D PC AVFF measurements correlated worse with and had a larger median difference from VOL than AIM_stat_ calculated using 4DF CMR static AVFF. Prior work using 4DF CMR and retrospective valve tracking annular inflow methods do not report their calculated AVVR as outcomes, instead comparing net inlet and outlet flows for internal validation, which limits direct comparison to our results as well as general clinical applicability [22, 25]. Recent work has demonstrated feasibility of an automatic valve-tracking 4DF CMR technique with improved reliability and internal consistency in measuring net valve flow across all four cardiac valves compared to manual valve-tracking technique in a large cohort of subjects with CHD [36]. However, their RF measurements were not compared to a clinical standard (such as VOL) or outcome measures. Future work assessing the accuracy of the automatic valve-tracking AIM method may demonstrate improved accuracy than found in our study with manual valve-tracking.

### Logistical advantages of 4DF CMR

2D CMR requires technical expertise from technologists and physicians to prescribe AV planes during the acquisition in patients with complex CHD anatomy, resulting in scan durations of 1–1.5 h [41, 53]. This has important implications on anesthetic requirements in pediatrics and on clinical efficiency. 4DF CMR, as a single acquisition with all planes created during post-processing, takes ~ 10 min after an initial localizer sequence. Acquisition of standard 2D PC requires multiple localizing sequences to achieve optimal orthogonal planes. Children are more likely to move in-between sequences, requiring repeat localizer sequences and longer exam times. This has resulted in the adoption of 4DF CMR for acquisition of all flow measurements at our institution, though 2D cine acquisitions are still necessary for planimetry.

Our post-processing analysis times for the AIM and JET methods were short and represent an opportunity to improve clinical flow and resource utilization in the MRI workflow. Our analysis time for the AIM_track_ method was significantly shorter than prior reports of 15–35 min per patient, likely due to the use of semi-automated software that adjusted to through plane motion [23, 25]. The JET method analysis time was relatively short (3.1 ± 2.1 min) irrespective of number and morphology of regurgitant jets.

### Limitations

Several limitations were present in our study. VOL in this study utilized 2D planimetry measurements and 4DF PC semilunar flow measurements, which precludes comparison of a true 2D VOL measurement to the 4DF JET. However, 4DF PC measurements of semilunar flow have been previously validated with excellent internal consistency and accuracy compared to 2D PC CMR [22, 30, 31, 35, 39], thus we do not believe this negatively impacted the validity of our results. We excluded subjects with significant semilunar regurgitation and our data may not be generalizable to those populations. Arterial encoding speeds were utilized in our cohort as part of our institution’s clinical practice based off prior published findings demonstrating reliable quantification of venous blood flow at arterial VENCs with the use of IV contrast, although this may have contributed to underestimation of the AIM method [41, 54]. It is not our clinical practice to perform multiple 4DF sequences at different VENC settings, though this may allow for more accurate data. Future application of phase unwrapping software may allow for a single, low-VENC 4DF sequence. Despite arterial VENCs, aliasing of regurgitant jets was common. However, tracing the jets distal to the aliasing segment allowed for accurate and reproducible data. We employ contrast agents for all our 4DF acquisitions. Recent research has found dose-dependent retention of gadolinium-based contrast in the brains of patients despite normal renal function, though long-term effects from chronic exposure remain unknown [55]. Although ferumoxytol is used clinically at an increasing number of centers for cardiac imaging, there remains a black box warning in pediatric imaging due to the small risk of adverse reactions, most notably hypotension [56]. Further study is necessary to clarify the risk:benefit ratio of contrast administration for these studies. Although our methodology for calculating AVVR differed slightly from published recommendations we accounted for even a small amount of semilunar regurgitation in order to optimize accuracy when comparing methodologies [13, 16, 53, 57]. Lastly, this study did not validate direct AVVR quantification against clinical outcome measures, as this was beyond the scope of the study.

## Conclusion

In a cohort of pediatric CHD, our findings support direct measurement of the regurgitant jet by 4DF CMR as an accurate and reliable method of measuring AVVR. In our cohort, the JET method was a superior method of internal validation to the VOL method than the AIM method by static- or valve-tracking- planes. We recommend at least 2 methods to measure AVVR. This redundancy exemplifies the benefits of 4DF CMR, and lends credence to the reported data. The JET method performed well in this heterogenous group, many of which had multiple, eccentric regurgitant jets. The slightly superior performance seen in subjects with tricuspid regurgitation compared to those with mitral regurgitation may be due to particularly eccentric or dynamic mitral jets. Future research should focus on validating our results in a larger, multicenter cohort of pediatric patients with CHD as well as correlation with outcomes including exercise capacity, development of ventricular dysfunction, surgical intervention, and mortality.

## Supplementary information

**Additional file 1.** 4DF CMR on Arterys of the AIM_track_ method in a patient with tricuspid regurgitation.

**Additional file 2.** 4DF CMR on Arterys of the JET method in a patient with mitral regurgitation.

## Data Availability

The data sets used and/or analyzed during the current study are available from the corresponding author on reasonable request.

## Abbreviations

4DF 4D flow AIM_stat_: Annular inflow method by static plane
AIM_track_: Annular inflow method by valve-tracking plane
AV: Atrioventricular valve
AVFF: Atrioventricular valve forward flow
AVSD: Atrioventricular septal defect
AVVR: Atrioventricular valve regurgitation
BA: Bland-Altman
bSSFP: Balanced steady-state free-precession
CHD: Congenital heart disease
CI: Confidence interval
CMR: Cardiovascular magnetic resonance
EDV: End-diastolic volume
EDVi: End-diastolic volume index
ESV: End-systolic volume
ESVi: End-systolic volume index
ICC: Intra-class correlation coefficient
IQR: Interquartile range
JET: Direct measurement of AVVR jet
LOA: Limits of agreement
LV: Left ventricle/left ventricular
MR: Mitral regurgitation
PC: Phase contrast
RF: Regurgitant fraction
RV: Right ventricle/right ventricular
RVol: Regurgitant volume
SD: Standard deviation
SLNF: Semilunar valve net flow
SV: Stroke volume
TR: Tricuspid regurgitation
VENC: Velocity encoding
VOL: Volumetric method
